# *In vitro* activity of apramycin (EBL-1003) in combination with colistin, meropenem, minocycline or sulbactam against XDR/PDR *Acinetobacter baumannii* isolates from Greece

**DOI:** 10.1093/jac/dkae077

**Published:** 2024-03-19

**Authors:** I Galani, M Souli, D Katsala, I Karaiskos, H Giamarellou, A Antoniadou

**Affiliations:** Infectious Diseases Laboratory, 4th Department of Internal Medicine, National and Kapodistrian University of Athens, School of Medicine, University General Hospital ‘ATTIKON’, Rimini 1, 124 62 Chaidari, Athens, Greece; Duke Clinical Research Institute, Duke University Medical Center, Durham, NC, USA; Infectious Diseases Laboratory, 4th Department of Internal Medicine, National and Kapodistrian University of Athens, School of Medicine, University General Hospital ‘ATTIKON’, Rimini 1, 124 62 Chaidari, Athens, Greece; 1st Department of Internal Medicine & Infectious Diseases, Hygeia General Hospital, Athens, Greece; 1st Department of Internal Medicine & Infectious Diseases, Hygeia General Hospital, Athens, Greece; Infectious Diseases Laboratory, 4th Department of Internal Medicine, National and Kapodistrian University of Athens, School of Medicine, University General Hospital ‘ATTIKON’, Rimini 1, 124 62 Chaidari, Athens, Greece; University of Cyprus, Medical School, Nicosia, Cyprus

## Abstract

**Objectives:**

To evaluate the *in vitro* activity of the combination of apramycin with colistin, meropenem, minocycline or sulbactam, against some well-characterized XDR *Acinetobacter baumannii* clinical isolates from Greece, to understand how apramycin can be best incorporated into clinical practice and optimize effectiveness.

**Methods:**

*In vitro* interactions of apramycin (0.5×, 1× and 2× the MIC value) with colistin (2 mg/L), meropenem (30 mg/L), minocycline (3.5 mg/L) or sulbactam (24 mg/L) were tested using time–kill methodology. Twenty-one clinical *A. baumannii* isolates were chosen, exhibiting apramycin MICs of 4–16 mg/L, which were at or below the apramycin preliminary epidemiological cut-off value of 16 mg/L. These isolates were selected for a range of colistin (4–32 mg/L), meropenem (16–256 mg/L), minocycline (8–32 mg/L) and sulbactam (8–32 mg/L) MICs across the resistant range. Synergy was defined as a ≥2 log_10_ cfu/mL reduction compared with the most active agent.

**Results:**

The combination of apramycin with colistin, meropenem, minocycline or sulbactam was synergistic, at least at one of the concentrations of apramycin (0.5×, 1× or 2× MIC), against 83.3%, 90.5%, 90.9% or 92.3% of the tested isolates, respectively. Apramycin alone was bactericidal at 24 h against 9.5% and 33.3% of the tested isolates at concentrations equal to 1× and 2× MIC, while the combination of apramycin at 2× MIC with colistin, meropenem or sulbactam was bactericidal against all isolates tested (100%). The apramycin 2× MIC/minocycline combination had bactericidal activity against 90.9% of the tested isolates.

**Conclusions:**

Apramycin combinations may have potential as a treatment option for XDR/pandrug-resistant (PDR) *A. baumannii* infections and warrant validation in the clinical setting, when this new aminoglycoside is available for clinical use.

## Introduction

*Acinetobacter baumannii* is an important cause of healthcare-associated infections, particularly in ICUs,^1^ known for its ability to acquire resistance to several antimicrobial agents.^2^ Most common *A. baumannii* infections consist of bloodstream infections (BSIs) and hospital-acquired pneumonia (HAP), including ventilator-associated pneumonia (VAP).^3^

Recent data from the European Antimicrobial Resistance Surveillance Network (EARS-Net) show a large increase of 57% in *Acinetobacter* spp. BSI in the EU and European Economic Area (EEA) in the first years of the COVID-19 pandemic (2020–21) compared with 2018–19.^4^ Additionally, among isolates from ICUs in countries with high (≥50%) prevalence of carbapenem-resistant *Acinetobacter* spp., carbapenem resistance increased from 73.2% to 85.6% over this 3 year period.^4^

Due to the lack of effective treatment options, infections caused by carbapenem-resistant *A. baumannii* (CR-Ab) are difficult to treat, with high mortality rates.^5^ The WHO has designated CR-Ab as a priority pathogen with an urgent need for additional research and development of new antibiotics.^6^ Apramycin (EBL-1003) has been proposed as a possible next-generation aminoglycoside, and it is currently the only new aminoglycoside in clinical development for the treatment of Gram-negative systemic infections.^7,8^ Based on its unique chemical structure, comprising an unusual bicyclic octose moiety, apramycin evades almost all clinically relevant aminoglycoside-modifying enzymes (AMEs) and is also unaffected by 16S rRNA-methyltransferase (RMTase)—mediated pan-aminoglycoside resistance.^9^

Moreover, although apramycin is not available for human use yet, it has been used in veterinary infectious diseases since the 1980s and rare apramycin resistance has been reported due to *aac(3)-IV*.^9^

A Phase I trial has reported that apramycin in healthy volunteers is ‘safe and well tolerated’ and the PK/PD modelling studies predicted an 8–16 mg/L target attainment at a human dose of 30 mg/kg.^10,11^

A few studies have evaluated the *in vitro* activity of apramycin against *A. baumannii* isolates and showed that is highly active against MDR, XDR and pandrug-resistant (PDR) clinical isolates.^9,12–16^ In time–kill studies, apramycin demonstrated rapid bactericidal activity within 1–2 h of antibiotic exposure at 2× and 4× MIC in three *A. baumannii* isolates with broth microdilution (BMD) MICs of 2, 16 and 64 mg/L, while antibiotic exposure at 1× MIC demonstrated rapid bactericidal activity only in the isolate with an MIC of 64 mg/L.^17^

In the present study, the *in vitro* activity of the combination of apramycin with colistin, meropenem, minocycline or sulbactam, against some well-characterized XDR *A. baumannii* clinical isolates from Greece was evaluated, to understand how apramycin can be best incorporated into clinical practice and optimize effectiveness.

## Materials and methods

### Bacteria

Strains used in this study were collected from among 271 *A. baumannii* blood isolates that were sent to our laboratory from 19 hospitals located in all seven Health Districts of Greece, from November 2020 to April 2021, during a multicentre surveillance study.^16^ Identification and MIC determination were performed using the VITEK 2 system (bioMérieux, Marcy-l’Étoile, France). MICs of apramycin, colistin, meropenem, minocycline and sulbactam were determined by the BMD method and results were interpreted in accordance with EUCAST.^18,19^  *Escherichia coli* ATCC 25922, *Pseudomonas aeruginosa* ATCC 27853 and *E. coli* NCTC 13846 (*mcr-1* positive) were used as quality control (QC) strains. Detection of antimicrobial resistance genes encoding common class D carbapenemases (*bla*_OXA-51_-like, *bla*_OXA-58_-like, *bla*_OXA-23_-like, *bla*_OXA-40_-like, *bla*_OXA-143_-like and *bla*_OXA-235_-like), class B MBLs (*bla*_IMP_, *bla*_VIM_ and *bla*_NDM_), RMTase genes (*armA*, *rmtB* and *rmtC*), AME genes [*aph(3′)-VI*, *aac(6′)-Ib*, *aac(3′)-Ia*, *aac(3′)-IV* and *ant(2′)-Ia*] and plasmid-mediated colistin resistance genes were screened by multiplex or simplex PCR protocols as described previously.^16^

### Time–kill studies

#### Static-concentration time–kill experiments

*In vitro* interactions of apramycin with colistin, meropenem, minocycline or sulbactam were tested using time–kill methodology. Twenty-one clinical *A. baumannii* isolates were chosen, exhibiting apramycin MICs of 4–16 mg/L, which were at or below the apramycin preliminary epidemiological cut-off (ECOFF) value of 16 mg/L proposed by Juhas *et al*.^9^ These isolates were selected for a range of colistin (4–32 mg/L), meropenem (16–32 mg/L), minocycline (8–32 mg/L) and sulbactam (8–32 mg/L) MICs across the resistant range. Overall, 3, 11 and 7 isolates with apramycin MICs of 16, 8 and 4 mg/L, respectively, were included in time–kill studies, to determine whether synergy was observed with colistin, meropenem, minocycline or sulbactam to which the isolates were resistant. Eight of the 21 isolates exhibiting meropenem MICs of >32 (64–256 mg/L), were also tested for synergy with apramycin/meropenem combination (Table 1).

**Table 1.** Characteristics of the 21 blood *A. baumannii* isolates used in this study

Strain number	MIC (mg/L)					Indication of MDR status	Genes coding for carbapenemases, β-lactamases and aminoglycoside resistance	Typing^a^
	APR	CST	MEM	SUL	MIN			
AC-381	16	64	64	>32	>32	PDR	*bla*_OXA-23_, *bla*_TEM_, *armA*	G1/ICII
AC-416	16	8	256	>32	>32	PDR	*bla*_OXA-23_, *bla*_TEM_, *armA*	G1/ICII
AC-391	16	2	32	>32	16	XDR	*bla*_OXA-23_, *bla*_TEM_, *aph(3′)-VI*	G1/ICII
AC-760	8	64	64	32	16	PDR	*bla*_OXA-23_, *armA*	G1/ICII
AC-467	8	64	32	32	8	PDR	*bla*_OXA-23_, *bla*_TEM_, *armA*	G1/ICII
AC-609	8	64	32	32	8	PDR	*bla*_OXA-23_, *bla*_TEM_, *armA*	G1/ICII
AC-531	8	32	128	>32	16	PDR	*bla*_OXA-23_, *bla*_TEM_, *armA*	G5
AC-312	8	32	32	32	8	PDR	*bla*_OXA-23_, *armA*	G1/ICII
AC-709	8	4	64	>32	>32	PDR	*bla*_OXA-23_, *bla*_TEM_, *armA*	G1/ICII
AC-425	8	2	32	32	8	XDR	*bla*_OXA-23_, *aph(3′)-VI*	G1/ICII
AC-205	8	2	32	16	0.5	XDR	*bla*_OXA-23_, *bla*_TEM_, *armA*, *aph(3′)-VI*, *aac(3)-I*	G6
AC-224	8	2	16	16	0.5	XDR	*bla*_OXA-23_, *armA*, *aph(3′)-VI*, *aac(3)-I*	G6
AC-215	8	1	256	>32	0.25	XDR	*bla*_OXA-23_, *bla*_NDM_, *aph(3′)-VI*	G5
AC-682	8	1	128	>32	8	XDR	*bla*_OXA-23_, *bla*_NDM_, *aph(3′)-VI*, *aac(3)-I*	G2/ICI
AC-100	4	64	16	>32	1	XDR	*bla*_OXA-23_, *bla*_TEM_, *armA*, *aph(3′)-VI*	G1/ICII
AC-708	4	16	64	32	32	PDR	*bla*_OXA-23_, *bla*_TEM_, *armA*	G1/ICII
AC-52	4	4	32	16	32	PDR	*bla*_OXA-23_, *armA*, *aph(3′)-VI*, *aac(6′)-Ib*, *aac(3)-I*	G1/ICII
AC-54	4	4	16	32	0.5	XDR	*bla*_OXA-23_, *aac(3)-I*	G5
AC-268	4	2	32	8	0.5	XDR	*bla*_OXA-23_, *bla*_TEM_, *armA*	G6
AC-482	4	2	16	16	0.5	XDR	*bla*_OXA-23_, *aph(3′)-VI*, *aac(3)-I*	G2/ICI
AC-674	4	1	16	16	16	XDR	*bla*_OXA-23_, *armA*	G1/ICII

APR, apramycin; CST, colistin; MEM, meropenem; SUL, sulbactam; MIN, minocycline.

^a^Sequence group/clonal lineages.^14^

Apramycin was tested at concentrations of 0.5×, 1× and 2× the MIC value. Colistin was tested at a concentration of 2 mg/L (target steady-state colistin concentration when initiating therapy, achieved by 12 h dosing schedule).^20^ Meropenem was tested at a concentration of 30 mg/L, simulating the *C*_max_ of a prolonged (3 h) infusion regimen of 1 g.^21^ Minocycline and sulbactam were tested at 3.5 and 24 mg/L, respectively, which represent the *C*_max_ of a 200 mg oral single-dose tablet for minocycline and 1.0 g for three consecutive IV doses for sulbactam.^22,23^

Tubes containing CAMHB (Becton Dickinson & Co., Sparks, MD, USA) and each antibiotic alone or in combination were inoculated with 10^5^ cfu/mL of the studied strain and were quantitatively subcultured at 0, 1, 3, 5 and 24 h of incubation for viable colony counts. At the above time intervals, an aliquot of 0.1 mL was removed from each tube, serially diluted (seven 10-fold dilutions) and plated on MacConkey agar (Becton Dickinson) plates. Dilution was expected to minimize any probable antibiotic carry-over effect. The results were expressed as log_10_ cfu/mL. A tube without antibiotic was also included in each experiment as a growth control. The lower limit of detection was 1.6 log_10_ cfu/mL.

Synergy was defined as a ≥2 log_10_ decrease in cfu/mL between the combination and the most active single agent at 24 h, with the number of surviving organisms in the presence of the combination being at least 2 log_10_ cfu/mL below the number of organisms in the starting inoculum. Antagonism was defined as a ≥2 log_10_ increase in cfu/mL between the combination and the most active single agent. All other interactions were characterized as indifferent. The bactericidal activity of single antibiotics or combinations was defined as a ≥3 log_10_ reduction in the cfu/mL of the initial inoculum. All studies were conducted in duplicate and the combined data are presented as mean bacterial density (cfu/mL) for all isolates.

## Results

MICs of apramycin, colistin, meropenem, minocycline and sulbactam, as determined by the BMD method, are presented in Table 1. Changes in bacterial density from 0 to 24 h for apramycin 0.5×, 1× and 2× the MIC value, colistin 2, meropenem 30, minocycline 3.5 and sulbactam 24 mg/L alone and in combination, for each isolate tested, are presented in Tables 2 –5.

**Table 2.** Interactions of apramycin/colistin combinations and differences between the starting inoculums and the number of residual viable colonies (Δlog_10_ cfu/mL) after 24 h of incubation with each combination

Strain	MIC (mg/L)		Interaction of combination at 24 h			Δlog_10_ cfu/mL between initial and final inoculum^a^						
			APR/COL			COL	APR			APR/COL		
	APR	COL	0.5×	1×	2×		0.5×	1×	2×	0.5×	1×	2×
AC-381	16	64	**SYN**	**SYN**	IND^b^	3.48	2.85	2.30	**−4**.**00**	**−4**.**30**	**−4**.**00**	**−4**.**30**
AC-416	16	8	**SYN**	**SYN**	**SYN**	1.57	2.12	3.30	0.10	**−4**.**70**	**−4**.**00**	**−4**.**00**
AC-760	8	64	IND	IND	**SYN**	2.63	1.16	1.06	1.94	1.82	0.66	**−4**.**81**
AC-467	8	64	IND	**SYN**	**SYN**	1.70	1.90	2.32	1.46	1.57	**−4**.**96**	**−4**.**96**
AC-609	8	64	IND	IND	**SYN**	2.15	2.30	2.61	1.32	1.27	0.78	**−4**.**30**
AC-531	8	32	**SYN**	**SYN**	**SYN**	1.91	2.39	2.34	−1.24	**−4**.**96**	**−4**.**70**	**−4**.**30**
AC-312	8	32	**SYN**	**SYN**	**SYN**	2.70	3.09	2.80	1.92	**−4**.**60**	**−4**.**30**	**−4**.**48**
AC-709	8	4	IND^c^	IND^c^	IND^c^	**−4**.**60**	2.08	3.00	**−4**.**48**	**−4**.**78**	**−4**.**30**	**−4**.**00**
AC-100	4	64	IND	**SYN**	**SYN**	2.85	2.52	2.48	2.71	1.46	−1.18	**−4**.**74**
AC-708	4	16	IND	**SYN**	**SYN**	3.11	2.52	2.78	1.70	2.10	**−4**.**00**	**−4**.**15**
AC-52	4	4	**SYN**	**SYN**	**SYN**	2.00	2.60	1.80	−1.17	**−4**.**85**	**−4**.**78**	**−5**.**00**
AC-54	4	4	IND^c^	IND^c^	IND^c^	**−4**.**88**	2.24	1.90	1.33	**−4**.**30**	**−4**.**00**	**−4**.**00**
Total *n* with synergy at 24 h			5/12	8/12	9/12							
Total *n* with bactericidal activity						2/12	0/12	0/12	2/12	7/12	9/12	12/12

APR, apramycin; SYN, synergy; IND, indifference. 0.5×, 1×, 2×, apramycin concentration expressed as 0.5-, 1- or 2-fold the MIC; COL, colistin 2 mg/L.

^a^A negative sign denotes a reduction of inoculum compared with time 0; values in bold are consistent with bactericidal activity.

^b^Apramycin at the concentration used was bactericidal after 24 h.

^c^Colistin at the concentration used was bactericidal after 24 h.

**Table 3.** Mean difference between viable counts in the presence of the combination and viable counts in the presence of each antibiotic alone at different timepoints (Δlog_10_ cfu/mL)

Antibiotic combination versus each antibiotic alone	Δlog_10_ cfu/mL at different timepoints			
	1 h	3 h	5 h	24 h
APR 0.5× + CST versus APR	−0.44	**−3.91**	**−5**.**04**	**−6**.**65**
APR 0.5× + CST versus CST	−0.02	−1.90	**−2**.**25**	**−6**.**38**
APR 1× + CST versus APR	−0.95	**−2**.**64**	−1.99	**−6**.**41**
APR 1× + CST versus CST	−0.53	**−2**.**30**	**−2**.**45**	**−6**.**08**
APR 2× + CST versus APR	−0.83	−1.34	−0.73	**−5**.**63**
APR 2× + CST versus CST	−1.04	**−2**.**60**	**−2**.**45**	**−6**.**38**
APR 0.5× + MEM versus APR	−0.10	**−2**.**86**	**−4**.**65**	−1.39
APR 0.5× + MEM versus MEM	−0.10	**−3**.**52**	**−5**.**27**	−1.25
APR 1× + MEM versus APR	0.00	−1.70	−1.55	**−6**.**61**
APR 1× + MEM versus MEM	−0.18	**−4**.**22**	**−5**.**28**	**−6**.**43**
APR 2× + MEM versus APR	−0.56	−1.00	−0.34	**−5**.**09**
APR 2× + MEM versus MEM	−1.00	**−4**.**90**	**−5**.**28**	**−6**.**60**
APR 0.5× + MIN versus APR	−0.43	−1.13	**−2**.**15**	**−3**.**93**
APR 0.5× + MIN versus MIN	−0.52	−1.80	**−2**.**85**	**−3**.**11**
APR 1× + MIN versus APR	0.39	−0.42	−0.89	**−5**.**85**
APR 1× + MIN versus MIN	−0.12	**−3**.**24**	**−4**.**87**	**−5**.**52**
APR 2× + MIN versus APR	−0.10	−0.61	−0.48	**−4**.**00**
APR 2× + MIN versus MIN	−0.80	**−5**.**09**	**−5**.**60**	**−5**.**78**
APR 0.5× + SUL versus APR	−0.24	**−4**.**12**	**−5**.**10**	**−6**.**66**
APR 0.5× + SUL versus SUL	−0.24	**−3**.**03**	**−3**.**20**	**−6**.**22**
APR 1× + SUL versus APR	−0.09	**−2**.**48**	−1.57	**−6**.**41**
APR 1× + SUL versus SUL	−0.30	**−3**.**42**	**−3**.**22**	**−6**.**13**
APR 2× + SUL versus APR	−0.32	−0.72	−0.50	**−5**.**79**
APR 2× + SUL versus SUL	−0.73	**−3**.**63**	**−3**.**43**	**−6**.**22**

Each value represents the average of values calculated for that timepoint for *A. baumannii* isolates using time–kill studies. A negative sign denotes a reduction of viable counts in the presence of the combination compared with counts in the presence of antibiotic alone; values in bold are consistent with a ≥2 log_10_ reduction.

APR 0.5×, apramycin (EBL-1003) at 0.5× MIC (mg/L); APR 1×, apramycin (EBL-1003) at 1× MIC (mg/L); APR 2×, apramycin (EBL-1003) at 2× MIC (mg/L); CST, colistin; MEM, meropenem; MIN, minocycline; SUL, sulbactam.

**Table 4.** Interactions of apramycin/meropenem combinations and differences between the starting inoculums and the number of residual viable colonies (Δlog_10_** **cfu/mL) after 24 h of incubation with each combination

Strain	MIC (mg/L)		Interaction of combination at 24 h			Δlog_10_ cfu/mL between initial and final inoculum^a^						
			APR/MEM			MEM	APR			APR/MEM		
	APR	MEM	0.5×	1×	2×		0.5×	1×	2×	0.5×	1×	2×
AC-416	16	256	IND	**SYN**	**SYN**	1.88	2.12	3.30	0.10	1.82	**−4**.**48**	**−4**.**48**
AC-381	16	64	IND	**SYN**	IND^b^	3.60	2.85	2.30	**−4**.**00**	2.48	**−3**.**91**	**−4**.**60**
AC-391	16	32	IND	IND^b^	IND^b,c^	1.22	1.40	**−4**.**30**	**−4**.**85**	2.06	**−4**.**30**	**−4**.**70**
AC-215	8	256	IND	**SYN**	IND^b^	1.90	1.67	1.15	**−4**.**48**	1.97	**−2**.**78**	**−4**.**70**
AC-531	8	128	IND	**SYN**	**SYN**	2.12	2.39	2.34	−1.24	1.60	**−4**.**00**	**−4**.**78**
AC-682	8	128	IND	**SYN**	IND^b^	3.00	3.00	**−2**.**60**	**−4**.**60**	2.70	**−4**.**30**	**−4**.**00**
AC-709	8	64	IND	IND	IND^b^	3.11	2.08	3.00	**−4**.**48**	2.30	2.10	**−4**.**70**
AC-760	8	64	IND	IND	**SYN**	2.15	1.16	1.06	1.94	1.52	3.24	**−4**.**80**
AC-205	8	32	**SYN**	**SYN**	**SYN**	3.10	1.70	1.71	1.93	**−4**.**00**	**−4**.**00**	**−4**.**00**
AC-312	8	32	**SYN**	**SYN**	**SYN**	2.22	3.09	2.80	1.92	**−4**.**85**	**−4**.**30**	**−4**.**78**
AC-425	8	32	IND	**SYN**	**SYN**	2.00	0.10	1.13	−0.30	1.70	**−4**.**30**	**−4**.**00**
AC-467	8	32	IND	**SYN**	**SYN**	2.06	1.90	2.32	1.46	0.88	**−4**.**48**	**−4**.**48**
AC-609	8	32	**SYN**	**SYN**	**SYN**	1.92	2.30	2.61	1.32	−1.00	**−4**.**60**	**−4**.**00**
AC-224	8	16	**SYN**	**SYN**	IND^b^	2.34	3.00	1.24	**−4**.**00**	**−4**.**60**	**−4**.**48**	**−4**.**70**
AC-708	4	64	IND	**SYN**	**SYN**	3.29	2.52	2.78	1.70	1.24	**−4**.**70**	**−4**.**48**
AC-52	4	32	**SYN**	**SYN**	**SYN**	2.12	2.60	1.80	−1.17	**−4**.**48**	**−5**.**00**	**−3**.**98**
AC-268	4	32	**SYN**	**SYN**	**SYN**	2.51	3.48	3.00	2.60	**−4**.**30**	**−4**.**30**	**−4**.**30**
AC-54	4	16	**SYN**	**SYN**	**SYN**	2.22	2.24	1.90	1.33	**−4**.**81**	**−4**.**55**	**−4**.**66**
AC-100	4	16	IND	**SYN**	**SYN**	1.85	2.52	2.48	2.71	0.00	**−3**.**88**	**−4**.**00**
AC-482	4	16	**SYN**	**SYN**	**SYN**	1.98	2.08	2.51	1.13	**−4**.**60**	**−4**.**00**	**−4**.**60**
AC-674	4	16	**SYN**	IND^b^	IND^b^	1.70	2.00	**−4**.**60**	**−4**.**30**	**−4**.**70**	**−4**.**30**	**−3**.**78**
Total *n* with synergy at 24 h			9/21	17/21	14/21							
Total *n* with bactericidal activity						0/21	0/21	2/21	7/21	8/21	18/21	21/21

APR, apramycin; 0.5×, 1×, 2×, apramycin concentration expressed as 0.5-, 1- or 2-fold the MIC; MEM, meropenem 30 mg/L; SYN, synergy; IND, indifference.

^a^A negative sign denotes a reduction of inoculum compared with time 0; values in bold are consistent with bactericidal or bacteriostatic activity.

^b^Apramycin at the concentration used was bactericidal after 24 h.

^c^ A reduction in the viable counts of bacterial cells of ≥2 log_10_ cfu/mL was observed with the combination after 1 h, but apramycin was bactericidal at 3–24 h.

**Table 5.** Interactions of apramycin/minocycline combinations and differences between the starting inoculums and the number of residual viable colonies (Δlog_10_ cfu/mL) after 24 h of incubation with each combination

Strain	MIC (mg/L)		Interaction of combination at 24 h			Δlog_10_ cfu/mL between initial and final inoculum^a^						
			APR/MIN			MIN	APR			APR/MIN		
	APR	MIN	0.5×	1×	2×		0.5×	1×	2×	0.5×	1×	2×
AC-391	16	16	**SYN**	IND^b^	IND^b^	1.70	1.40	**−4**.**30**	**−4**.**85**	**−5**.**06**	**−4**.**70**	**−4**.**48**
AC-531	8	16	IND	**SYN**	**SYN**	1.12	2.39	2.34	−1.24	1.05	**−4**.**00**	**−4**.**48**
AC-760	8	16	IND	IND	**SYN**	1.76	1.16	1.06	1.94	0.95	1.07	**−4**.**96**
AC-312	8	8	**SYN**	**SYN**	**SYN**	0.60	3.09	2.80	1.92	−1.63	**−4**.**60**	**−4**.**30**
AC-425	8	8	IND	**SYN**	**SYN**	1.48	0.10	1.13	−0.30	0.40	**−4**.**04**	**−4**.**00**
AC-467	8	8	**SYN**	**SYN**	**SYN**	1.00	1.90	2.32	1.46	−1.78	**−4**.**00**	**−4**.**70**
AC-609	8	8	**SYN**	**SYN**	**SYN**	0.50	2.30	2.61	1.32	**−4**.**85**	**−4**.**30**	**−4**.**30**
AC-682	8	8	**SYN**	**SYN**	IND^b^	3.30	3.00	**−2**.**60**	**−4**.**60**	1.00	**−3**.**60**	**−4**.**48**
AC-52	4	32	**SYN**	**SYN**	**SYN**	1.92	2.60	1.80	−1.17	**−4**.**48**	**−4**.**30**	**−4**.**25**
AC-708	4	32	IND	IND	IND	2.48	2.52	2.78	1.70	1.37	0.88	1.48
AC-674	4	16	**SYN**	IND^b^	IND^b^	1.10	2.00	**−4**.**60**	**−4**.**30**	**−4**.**30**	**−4**.**30**	**−4**.**30**
Total *n* with synergy at 24 h			7/11	7/11	7/11							
Total *n* with bactericidal activity						0/11	0/11	2/11	3/11	4/11	9/11	10/11

APR, apramycin; 0.5×, 1×, 2×, apramycin concentration expressed as 0.5-, 1- or 2-fold the MIC; MIN, minocycline 3.5 mg/L; SYN, synergy; IND, indifference.

^a^A negative sign denotes a reduction of inoculum compared with time 0; values in bold are consistent with bactericidal or bacteriostatic activity.

^b^Apramycin at the concentration used was bactericidal after 24 h.

Apramycin alone was bactericidal at 24 h against two (9.5%) and seven (33.3%) tested isolates at concentrations equal to 1× and 2× MIC, respectively (Table 4), while at concentrations 4× and 8× MIC was bactericidal against 16 (76.2%) and all (100%) isolates (data not shown). Apramycin demonstrated rapid bactericidal activity within 3 h of antibiotic exposure at 2× MIC, exhibiting an average of Δlog_10_ cfu/mL between initial and final inoculum of −3.19, which was increased to −3.47 at 5 h but decreased at −0.73 at 24 h; Tables S1–S5 and Figure S1 (available as Supplementary data at *JAC* Online).

## Synergy testing by time–kill assays

### Apramycin/colistin

Twelve isolates were resistant to colistin and exhibited MICs higher than the EUCAST ECOFF of 2 mg/L, according to the BMD method (Table 1). The combination of apramycin with colistin at 2 mg/L was synergistic, at least at one of the concentrations of apramycin (0.5×, 1× or 2× MIC), against 10 of the 12 isolates tested (83.3%) (Table 2). In the two isolates exhibiting no synergy (AC-54 and AC-709), colistin at 2 mg/L alone was bactericidal, although the colistin MIC was 4 mg/L. The combination of apramycin at 0.5× MIC, 1× MIC or 2× MIC with colistin was synergistic against 5 (41.7%), 8 (66.7%) or 9 (75%) of the tested isolates (Table 2), exhibiting a decrease in log_10_ cfu/mL of 6.3–7.1 (median 6.5), 3.5–7.0 (median 6.5) or 3.3–6.8 (median 6.3), respectively, compared with the most active compound. The difference between the mean starting inoculum and the mean viable cell count (Δlog_10_ cfu/mL) over time of all *A. baumannii* isolates tested is presented in Figure S2. The combination of apramycin at 2× MIC with colistin had bactericidal activity against all isolates tested (100%).

### Apramycin/meropenem

All strains were resistant to meropenem, according to the BMD method [MIC values of all strains >16 mg/mL, (Table 1)]. The combination of apramycin with meropenem was synergistic, at least at one of the concentrations of apramycin (0.5×, 1× or 2× MIC), against 19 of the 21 isolates tested (90.5%) (Table 4). One of the two isolates exhibiting no synergy (AC-391), had an apramycin MIC of 16 mg/L. Bactericidal activity was observed with apramycin at 16 (1× MIC) and 32 mg/L (2× MIC) alone or with either of the combinations with meropenem. Apramycin at 8 mg/L (0.5× MIC) was synergistic with meropenem at 5 h but regrowth occurred at 24 h (data not shown). The second isolate (AC-709) exhibited a meropenem MIC of 64 mg/L and an apramycin MIC of 8 mg/L and no synergy or bactericidal/bacteriostatic activity was observed with the drugs alone or with either of the combinations with apramycin at 0.5× or 1× MIC. Bactericidal activity was observed for apramycin at 2× MIC alone and in combination with meropenem. The combination of apramycin at 0.5× MIC, 1× MIC or 2× MIC with meropenem was synergistic against 9 (42.9%), 17 (81.0%) or 14 (66.7%) of the tested isolates (Table 4), exhibiting a decrease in log_10_ cfu/mL of 3.1–7.0 (median 6.6), 2.0–7.2 (median 6.5) or 3.3–6.8 (median 5.9), respectively, compared with the most active compound. The difference between the mean starting inoculum and the mean viable cell count (Δlog_10_ cfu/mL) over time of all *A. baumannii* isolates tested is presented in Figure S3. The combination of apramycin at 2× MIC with meropenem had bactericidal activity against all isolates tested (100%).

### Apramycin/minocycline

Eleven strains were resistant to minocycline according to the BMD method [MIC values 8–32 mg/mL, (Table 1)]. The combination of apramycin with minocycline was synergistic, at least at one of the concentrations of apramycin (0.5×, 1× or 2× MIC), against 10 out of 11 resistant isolates tested (90.9%), with the isolate exhibiting no synergy having a minocycline MIC of 32 mg/L. Minocycline at 3.5 mg/L was bactericidal against all isolates exhibiting MICs of ≤1 mg/L (data not shown). The combination of apramycin at 0.5× MIC, 1× MIC or 2× MIC with minocycline was synergistic against 7 (63.6%) of the tested isolates in all cases (Table 5), exhibiting a decrease in log_10_ cfu/mL of 2.3–7.0 (median 5.0), 2.0–6.3 (median 5.3) or 3.3–6.6 (median 5.3), respectively, compared with the most active compound. The difference between the mean starting inoculum and the mean viable cell count (Δlog_10_ cfu/mL) over time of all *A. baumannii* isolates tested is presented in Figure S4. The combination of apramycin at 2× MIC with minocycline had bactericidal activity against 10/11 isolates tested (90.9%).

### Apramycin/sulbactam

All isolates exhibited sulbactam MICs of ≥8 mg/L, according to the BMD method [MIC values 8–32 mg/mL, (Table 1)]. The combination of apramycin with sulbactam was synergistic, at least at one of the concentrations of apramycin (0.5×, 1× or 2× MIC), against 12/13 isolates tested (92.3%). The isolate exhibiting no synergy (AC-482), had a sulbactam MIC of 16 mg/L and a bactericidal activity was observed with sulbactam alone at 24 mg/L. The combination of apramycin at 0.5× MIC, 1× MIC or 2× MIC with sulbactam was synergistic against 10 (76.9%), 11 (84.6%) or 10 (76.9%) of the tested isolates (Table 6), exhibiting a decrease in log_10_ cfu/mL of 2.0–7.3 (median 6.4), 3.8–7.3 (median 6.6) or 3.6–6.9 (median 6.0), respectively, compared with the most active compound. The difference between the mean starting inoculum and the mean viable cell count (Δlog_10_ cfu/mL) over time of all *A. baumannii* isolates tested is presented in Figure S5. The combination of apramycin at 1× MIC or 2× MIC with sulbactam had bactericidal activity against all isolates tested (100%).

**Table 6.** Interactions of apramycin/sulbactam combinations and differences between the starting inoculums and the number of residual viable colonies (Δlog_10_ cfu/mL) after 24 h of incubation with each combination

Strain	MIC (mg/L)		Interaction of combination at 24 h			Δlog_10_ cfu/mL between initial and final inoculum^a^						
			APR/SUL			SUL	APR			APR/SUL		
	APR	SUL	0.5×	1×	2×		0.5×	1×	2×	0.5×	1×	2×
AC-312	8	32	**SYN**	**SYN**	**SYN**	2.98	3.09	2.80	1.92	**−4**.**85**	**−4**.**00**	**−4**.**70**
AC-425	8	32	**SYN**	**SYN**	**SYN**	0.88	0.10	1.13	−0.30	**−4**.**48**	**−4**.**30**	**−4**.**30**
AC-467	8	32	**SYN**	**SYN**	**SYN**	2.43	1.90	2.32	1.46	**−4**.**70**	**−4**.**78**	**−4**.**30**
AC-609	8	32	IND	**SYN**	**SYN**	2.30	2.30	2.61	1.32	1.48	**−4**.**78**	**−4**.**48**
AC-760	8	32	**SYN**	**SYN**	**SYN**	0.07	1.16	1.06	1.94	**−4**.**91**	**−4**.**30**	**−4**.**88**
AC-205	8	16	**SYN**	**SYN**	**SYN**	1.35	1.70	1.71	1.93	**−4**.**30**	**−4**.**30**	**−4**.**00**
AC-224	8	16	**SYN**	**SYN**	IND^b^	3.00	3.00	1.24	**−4**.**00**	**−4**.**60**	**−4**.**00**	**−4**.**48**
AC-54	4	32	**SYN**	**SYN**	**SYN**	2.60	2.24	1.90	1.33	**−4**.**78**	**−4**.**78**	**−4**.**60**
AC-708	4	32	IND	**SYN**	**SYN**	2.90	2.52	2.78	1.70	0.50	**−4**.**60**	**−4**.**48**
AC-52	4	16	**SYN**	**SYN**	**SYN**	−0.93	2.60	1.80	−1.17	**−4**.**00**	**−4**.**30**	**−3**.**91**
AC-482	4	16	IND^c^	IND^c^	IND^c^	**−4**.**00**	2.08	2.51	1.13	**−4**.**02**	**−4**.**06**	**−4**.**60**
AC-674	4	16	**SYN**	IND^b^	IND^b^	**−2**.**30**	2.00	**−4**.**60**	**−4**.**30**	**−4**.**30**	**−3**.**88**	**−3**.**70**
AC-268	4	8	**SYN**	**SYN**	**SYN**	2.57	3.48	3.00	2.60	**−4**.**48**	**−4**.**48**	**−4**.**70**
Total *n* with synergy at 24 h			10/13	11/13	10/13							
Total *n* with bactericidal activity						1/13	0/13	1/13	2/13	11/13	13/13	13/13

APR, apramycin; 0.5×, 1×, 2×, apramycin concentration expressed as 0.5-, 1- or 2-fold the MIC; SUL, sulbactam 24 mg/L; SYN, synergy; IND, indifference.

^a^A negative sign denotes a reduction of inoculum compared with time 0; values in bold are consistent with bactericidal or bacteriostatic activity.

^b^Apramycin at the concentration used was bactericidal/bacteriostatic after 24 h.

^c^Sulbactam at the concentration used was bactericidal after 24 h.

## Discussion

As a consequence of limited therapeutic options for the treatment of *A. baumannii* infections, clinicians often resort to antibiotic combinations, in the hope of improving the activity of available agents. The combination of an aminoglycoside with another agent might promote the permeabilizing effect and enhance the periplasmic target site penetration of the second agent used in combination.^24,25^ Apramycin, the only new aminoglycoside in clinical development for the treatment of Gram-negative systemic infections,^7,8^ retains significant activity against a broad spectrum of MDR and XDR clinical isolates, including CR-Ab, with the presence of various aminoglycoside resistance-conferring genes, such as AMEs and RMTase-coding genes, not adversely affecting the distribution of apramycin MICs.^9^

CR-Ab is a critical Gram-negative organism included in the WHO priority pathogen list, with no optimal therapeutic strategy for the management of related infections,^26^ with sulbactam, meropenem, minocycline, tigecycline and polymyxins, the last-resort antibiotics in recent decades, for critically ill patients.^27^ In a recent study, conducted in Greece, the dissemination of XDR and PDR *bla*_OXA-23_-*armA*-harbouring *A. baumannii* isolates, corresponding to international clone (IC) II (87.8%), was associated with a dramatic decrease of *in vitro* activity of colistin to only 15.5%, leaving minocycline as the most active agent, with 18.8% of the isolates exhibiting an MIC of ≤4 mg/L.^16^ Interestingly, a promising *in vitro* activity was verified for apramycin (EBL-1003), which exhibited MIC_50_/MIC_90_ of 4/8 mg/L and 100% susceptibility according to the preliminary ECOFF (16 mg/L) defined by Juhas *et al* for *A. baumannii.*^9,16^ However, aminoglycoside monotherapy can lead to unfavourable clinical outcomes due to rapid emergence of resistance, while nephrotoxicity is a serious concern with prolonged use.^28,29^

In this study, excellent synergy was observed for apramycin and colistin, meropenem, minocycline or sulbactam combinations against *A. baumannii* XDR and PDR isolates despite resistance of the tested isolates to the second agent. The combinations of apramycin and colistin and apramycin and sulbactam exhibited bactericidal activity at all tested concentrations of apramycin, against XDR and PDR *A. baumannii* isolates. The bactericidal activity began as early as 3 h of incubation and continued up until 24 h. The combinations with meropenem and minocycline exhibited bactericidal activity at 1× and 2× MIC concentrations of apramycin from 3 to 24 h.

Literature review on apramycin combinations has revealed that only two laboratory research papers are currently available. The first laboratory study of Brennan-Krohn and Kirby^30^ reported that apramycin/colistin combination was not synergistic against the PDR *Klebsiella pneumoniae* Nevada strain, which was an NDM-1-producing and colistin-resistant (MIC 16 mg/L) strain.^30^ In the second study, colistin/apramycin synergy was observed against 15.3% of carbapenemase-producing and colistin-resistant *K. pneumoniae* strains, whereas other strains had antagonistic (30.7%) or additive (54%) effects.^31^ Additionally, the meropenem/apramycin combination was synergistic against 52%, while an additive effect was observed against 31% of the isolates tested.^31^ In both studies, synergy testing was conducted with the chequerboard microdilution method.

This study is a laboratory study that investigates the use of apramycin in combination with colistin, meropenem, minocycline or sulbactam against XDR/PDR *A. baumannii* blood isolates, with the time–kill method.

Proposed therapeutic approaches for CR-Ab severe infections include treatment with combination of two *in vitro* active agents, with preferable combinations, an aminoglycoside or a polymyxin with high-dose ampicillin/sulbactam or high-dose tigecycline or high-dose minocycline or *in vitro* synergy testing for selection of a combination scheme, when two *in vitro* active agents are not available.^32^ In the case of PDR CR-Ab infections, cefiderocol is recommended, if available, or the triple-drug combination of colistin, high-dose meropenem and high-dose ampicillin/sulbactam.^32^ The recent ESCMID guidelines recommend double-agent combination therapy when CR-Ab is susceptible to more than one antibiotic,^33^ while conditionally recommending against cefiderocol due to low certainty of evidence.

Aminoglycosides are among the active *in vitro* antibiotics commonly suggested for combination therapy against severe and high-risk CR-Ab infections. Thus, the high rate of *in vitro* activity of apramycin against the XDR/PDR *armA*-harbouring A. *baumannii* isolates is an interesting feature of this agent.^16^ Although all *A. baumannii* strains (100%) were susceptible to apramycin, apramycin alone at 2× MIC exhibited bactericidal activity against only seven strains (33.3%) and only at 24 h. On the contrary, all (100%) colistin, meropenem and sulbactam combinations with apramycin at 2× MIC exhibited bactericidal activity at 24 h. The combination of apramycin (2× MIC) with minocycline was bactericidal at 24 h against 10 of the 11 strains tested (90.9%), with one of these isolates exhibiting a minocycline MIC of 32 mg/L. Synergy rates were high for all combinations, ranging from 83.3% (for colistin) to 92.3% (for sulbactam), while none of the apramycin combinations tested in this study was antagonistic towards the *A. baumannii* strains.

Overall, our results suggest that apramycin combinations may have potential as a treatment option for XDR and PDR *A. baumannii* infections and warrant validation in the clinical setting, when this new aminoglycoside is available for clinical use.

## Supplementary Material

dkae077_Supplementary_Data

## Supplementary data

Figures S1 to S5 and Tables S1 to S5 are available as Supplementary data at *JAC* Online.
