# Supplementary material for: In vitro activity of apramycin (EBL-1003) in combination with colistin, meropenem, minocycline or sulbactam against XDR/PDR Acinetobacter baumannii isolates from Greece
Source: J Antimicrob Chemother. 2024 Mar 19;79(5):1101–8. doi: 10.1093/jac/dkae077 (PMC11062935; doi:10.1093/jac/dkae077)
Supplement: dkae077_Supplementary_Data [file dkae077_supplementary_data.doc]

**Table S1. Difference between viable counts in the presence of apramycin at 0x (control), 0.25x, 0.5x, 1x and 2xMIC at different timepoints (Δlog_10_ cfu/mL).**

| **Isolate** | **Apramycin concentration** | **Δlog_10_cfu/mL between initial and final inoculum^a^,** | | | |
| --- | --- | --- | --- | --- | --- |
|  |  | **Hours of incubation (h)** | | | |
|  |  | 1 | 3 | 5 | 24 |
| **AC-381** | CONTROL | 0.78 | 1.95 | 2.30 | 4.48 |
|  | 0.25xMIC | 0.18 | 1.54 | 1.70 | 2.78 |
|  | 0.5xMIC | 0.00 | -0.70 | -2.00 | 2.85 |
|  | 1xMIC | -1.40 | **-4.10** | **-3.40** | 2.30 |
|  | 2xMIC | -2.15 | **-4.00** | **-4.00** | **-4.00** |
| **AC-416** | CONTROL | 0.85 | 2.60 | 3.00 | 3.60 |
|  | 0.25xMIC | 0.43 | -0.18 | -0.48 | 2.48 |
|  | 0.5xMIC | 0.18 | -1.30 | -1.30 | 2.12 |
|  | 1xMIC | 0.00 | -0.70 | -1.22 | 3.30 |
|  | 2xMIC | -1.30 | -1.30 | -1.76 | 0.10 |
| **AC-391** | CONTROL | -0.30 | 1.22 | 1.62 | 2.22 |
|  | 0.25xMIC | 0.30 | -1.43 | 0.10 | 1.70 |
|  | 0.5xMIC | 0.00 | **-3.52** | -1.70 | 1.40 |
|  | 1xMIC | -0.40 | **-3.82** | **-4.30** | **-4.30** |
|  | 2xMIC | -2.75 | **-4.85** | **-4.85** | **-4.85** |
| **AC-682** | CONTROL | 0.00 | 1.95 | 2.00 | 4.00 |
|  | 0.25xMIC | 0.30 | 1.40 | 1.60 | 3.18 |
|  | 0.5xMIC | -0.30 | 0.30 | -0.49 | 3.00 |
|  | 1xMIC | -0.60 | -1.70 | -2.60 | -2.60 |
|  | 2xMIC | -0.43 | -1.76 | **-3.00** | **-4.60** |
| **AC-709** | CONTROL | 0.60 | 2.78 | 2.60 | 3.78 |
|  | 0.25xMIC | -0.10 | 1.60 | 2.18 | 2.60 |
|  | 0.5xMIC | 0.20 | 1.11 | 1.78 | 2.08 |
|  | 1xMIC | 0.10 | -1.30 | -1.44 | 3.00 |
|  | 2xMIC | 0.12 | -1.63 | -2.35 | **-4.48** |
| **AC-531** | CONTROL | 0.18 | 1.34 | 2.12 | 3.26 |
|  | 0.25xMIC | -0.34 | 1.00 | 1.20 | 2.59 |
|  | 0.5xMIC | -0.30 | 0.23 | -0.22 | 2.39 |
|  | 1xMIC | -0.73 | -1.50 | -2.36 | 2.34 |
|  | 2xMIC | -0.70 | -2.37 | -2.07 | -1.24 |
| **AC-312** | CONTROL | 0.34 | 1.92 | 2.34 | 3.12 |
|  | 0.25xMIC | 0.41 | 1.96 | 2.02 | 3.25 |
|  | 0.5xMIC | 0.24 | 1.07 | 1.52 | 3.09 |
|  | 1xMIC | -0.06 | -1.42 | **-3.30** | 2.80 |
|  | 2xMIC | -0.45 | **-3.63** | **-3.63** | 1.92 |
| **AC-425** | CONTROL | 0.44 | 1.40 | 2.40 | 2.76 |
|  | 0.25xMIC | 1.08 | 2.10 | 2.60 | 1.85 |
|  | 0.5xMIC | 0.28 | 0.18 | 0.70 | 0.10 |
|  | 1xMIC | -0.55 | -2.70 | **-3.40** | 1.13 |
|  | 2xMIC | -0.46 | **-4.60** | **-4.60** | -0.30 |
| **AC-609** | CONTROL | 0.05 | 1.12 | 1.30 | 2.74 |
|  | 0.25xMIC | 0.20 | 1.42 | 1.50 | 3.30 |
|  | 0.5xMIC | 0.00 | 0.38 | 0.85 | 2.30 |
|  | 1xMIC | 0.10 | -1.97 | -2.68 | 2.61 |
|  | 2xMIC | -0.63 | **-3.70** | **-3.52** | 1.32 |
| **AC-205** | CONTROL | 0.48 | 1.70 | 2.48 | 3.00 |
|  | 0.25xMIC | 0.12 | 1.43 | 1.22 | 2.82 |
|  | 0.5xMIC | -0.12 | 0.18 | -0.97 | 1.70 |
|  | 1xMIC | -0.44 | -2.29 | **-3.29** | 1.71 |
|  | 2xMIC | -0.30 | **-4.00** | **-4.00** | 1.93 |
| **AC-224** | CONTROL | 0.30 | 1.95 | 2.28 | 3.00 |
|  | 0.25xMIC | 0.00 | 1.48 | 1.46 | 2.52 |
|  | 0.5xMIC | 0.60 | 0.29 | -1.52 | 3.00 |
|  | 1xMIC | -0.30 | **-4.00** | **-4.60** | 1.24 |
|  | 2xMIC | -0.05 | **-4.00** | **-4.00** | **-4.00** |
| **AC-467** | CONTROL | 0.30 | 1.60 | 1.70 | 3.00 |
|  | 0.25xMIC | 0.00 | 1.43 | 1.52 | 3.12 |
|  | 0.5xMIC | -0.10 | 0.50 | 0.60 | 1.90 |
|  | 1xMIC | -0.85 | -2.85 | -2.54 | 2.32 |
|  | 2xMIC | -0.85 | **-3.10** | **-4.24** | 1.46 |
| **AC-760** | CONTROL | -0.09 | 1.02 | 1.71 | 2.71 |
|  | 0.25xMIC | -0.79 | 0.71 | 1.44 | 1.89 |
|  | 0.5xMIC | -0.25 | 0.08 | 1.02 | 1.16 |
|  | 1xMIC | -0.51 | -0.51 | -1.68 | 1.06 |
|  | 2xMIC | -0.81 | -2.20 | -2.00 | 1.94 |
| **AC-215** | CONTROL | 0.32 | 2.15 | 2.23 | 3.11 |
|  | 0.25xMIC | 0.04 | 1.21 | 2.07 | 2.70 |
|  | 0.5xMIC | -0.10 | -0.48 | -1.41 | 1.78 |
|  | 1xMIC | 0.00 | -1.73 | -2.03 | 1.48 |
|  | 2xMIC | -0.10 | -2.47 | -2.99 | **-4.48** |
| **AC-100** | CONTROL | 0.19 | 1.85 | 1.52 | 3.00 |
|  | 0.25xMIC | 0.00 | 0.90 | 1.15 | 3.11 |
|  | 0.5xMIC | -0.20 | 1.18 | 1.52 | 2.52 |
|  | 1xMIC | 0.00 | -0.41 | -0.67 | 2.48 |
|  | 2xMIC | 0.14 | -1.51 | -1.79 | 2.71 |
| **AC-52** | CONTROL | 0.99 | 1.71 | 2.55 | 3.76 |
|  | 0.25xMIC | 0.56 | -0.28 | -0.85 | 2.62 |
|  | 0.5xMIC | -0.06 | -2.04 | **-3.25** | 2.60 |
|  | 1xMIC | 0.40 | **-4.48** | **-4.00** | 1.80 |
|  | 2xMIC | -1.61 | **-4.80** | **-4.80** | -1.17 |
| **AC-674** | CONTROL | 0.02 | 1.82 | 2.05 | 3.65 |
|  | 0.25xMIC | -0.43 | -0.82 | -2.46 | 2.00 |
|  | 0.5xMIC | -1.30 | **-3.82** | **-4.30** | 2.00 |
|  | 1xMIC | -2.30 | **-4.60** | **-4.60** | **-4.60** |
|  | 2xMIC | **-4.30** | **-4.30** | **-4.30** | **-4.30** |
| **AC-54** | CONTROL | -0.15 | 1.09 | 1.00 | 2.37 |
|  | 0.25xMIC | -0.21 | 0.93 | 1.12 | 2.12 |
|  | 0.5xMIC | 0.07 | 0.81 | 1.44 | 2.24 |
|  | 1xMIC | -0.43 | -1.16 | -2.28 | 1.90 |
|  | 2xMIC | 0.14 | **-4.11** | **-3.97** | 1.33 |
| **AC-482** | CONTROL | 0.12 | 1.56 | 1.81 | 3.22 |
|  | 0.25xMIC | 0.08 | 1.86 | 2.06 | 2.85 |
|  | 0.5xMIC | 0.38 | 1.38 | 2.11 | 2.08 |
|  | 1xMIC | 0.18 | -1.40 | -1.90 | 2.51 |
|  | 2xMIC | 0.19 | -2.86 | **-3.57** | 1.13 |
| **AC-708** | CONTROL | 0.22 | 0.60 | 1.22 | 3.37 |
|  | 0.25xMIC | 0.22 | 0.40 | 1.52 | 3.00 |
|  | 0.5xMIC | -0.18 | 0.00 | 1.52 | 2.52 |
|  | 1xMIC | 0.00 | -0.05 | -0.22 | 2.78 |
|  | 2xMIC | -0.30 | -2.82 | **-3.82** | 1.70 |
| **AC-268** | CONTROL | 0.40 | 2.00 | 2.00 | 3.54 |
|  | 0.25xMIC | 0.70 | 1.90 | 2.00 | 3.60 |
|  | 0.5xMIC | 0.70 | 1.78 | 2.00 | 3.48 |
|  | 1xMIC | 0.22 | -0.78 | -1.32 | 3.00 |
|  | 2xMIC | -0.22 | -2.85 | **-3.70** | 2.60 |

0.25xMIC, 0.5xMIC, 1xMIX, 2xMIC, apramycin concentration expressed as 0.25-, 0.5-, 1- or 2-fold the MIC

^a^A negative sign denotes a reduction of inoculum compared with time 0; values in bold are consistent with bactericidal activity

**Table S2.** Average difference between viable counts in the presence of apramycin at 0x (control), 0.25x, 0.5x, 1x and 2xMIC at different timepoints (Δlog_10_ cfu/mL). All isolates included (n=21).

| **Apramycin concentration** | **Δlog_10_cfu/mL at different timepoints^a^** | | | |
| --- | --- | --- | --- | --- |
|  | 1h | 3h | 5h | 24h |
| CONTROL | 0.26 | 1.52 | 1.74 | 3.27 |
| APR 0.25x | 0.13 | 0.70 | 0.65 | 2.76 |
| APR 0.5x | -0.08 | -0.10 | 0.15 | 2.49 |
| APR 1x | -0.28 | -1.84 | -2.14 | 1.41 |
| APR 2x | -0.85 | **-3.32** | **-3.71** | 0.57 |

APR, apramycin; 0.25x, 0.5x, 1x, 2x, apramycin concentration expressed as 0.25-, 0.5-, 1- or 2-fold the MIC

^a^A negative sign denotes a reduction of inoculum compared with time 0; values in bold are consistent with bactericidal activity

**Figure S1.** Average difference between viable counts in the presence of apramycin at 0x (control), 0.25x, 0.5x, 1x and 2xMIC at different timepoints (Δlog_10_ cfu/mL).

APR, apramycin; 0.25x, 0.5x, 1x, 2x, apramycin concentration expressed as 0.25-, 0.5-, 1- or 2-fold the MIC

**Table S3.** Average difference between viable counts in the presence of apramycin at 0x (control), 0.25x, 0.5x, 1x and 2xMIC at different timepoints (Δlog_10_ cfu/mL). Isolates with apramycin MIC of 16mg/L are included (n=3).

| **Apramycin concentration** | **Δlog_10_cfu/mL at different timepoints^a^** | | | |
| --- | --- | --- | --- | --- |
|  | 1h | 3h | 5h | 24h |
| CONTROL | 0.44 | 1.93 | 2.31 | 3.43 |
| APR 0.25x | 0.30 | -0.02 | 0.44 | 2.32 |
| APR 0.5x | 0.06 | -1.84 | -1.67 | 2.12 |
| APR 1x | -0.60 | -2.87 | -2.97 | 0.43 |
| APR 2x | -2.07 | **-3.38** | **-3.54** | -2.92 |

APR, apramycin; 0.25x, 0.5x, 1x, 2x, apramycin concentration expressed as 0.25-, 0.5-, 1- or 2-fold the MIC

^a^A negative sign denotes a reduction of inoculum compared with time 0; values in bold are consistent with bactericidal activity

**Table S4.** Average difference between viable counts in the presence of apramycin at 0x (control), 0.25x, 0.5x, 1x and 2xMIC at different timepoints (Δlog_10_ cfu/mL). Isolates with apramycin MIC of 8mg/L are included (n=11).

| **Apramycin concentration** | **Δlog_10_cfu/mL at different timepoints^a^** | | | |
| --- | --- | --- | --- | --- |
|  | 1h | 3h | 5h | 24h |
| CONTROL | 0.26 | 1.72 | 2.11 | 3.14 |
| APR 0.25x | 0.08 | 1.43 | 1.71 | 2.71 |
| APR 0.5x | 0.01 | 0.35 | 0.17 | 2.05 |
| APR 1x | -0.35 | -2.00 | -2.72 | 1.55 |
| APR 2x | -0.42 | **-3.04** | **-3.31** | -0.96 |

APR, apramycin; 0.25x, 0.5x, 1x, 2x, apramycin concentration expressed as 0.25-, 0.5-, 1- or 2-fold the MIC

^a^A negative sign denotes a reduction of inoculum compared with time 0; values in bold are consistent with bactericidal activity

**Table S5.** Average difference between viable counts in the presence of apramycin at 0x (control), 0.25x, 0.5x, 1x and 2xMIC at different timepoints (Δlog_10_ cfu/mL). Isolates with apramycin MIC of 4 mg/L are included (n=7).

| **Apramycin concentration** | **Δlog_10_cfu/mL at different timepoints^a^** | | | |
| --- | --- | --- | --- | --- |
|  | 1h | 3h | 5h | 24h |
| CONTROL | 0.26 | 1.52 | 1.74 | 3.27 |
| APR 0.25x | 0.13 | 0.70 | 0.65 | 2.76 |
| APR 0.5x | -0.08 | -0.10 | 0.15 | 2.49 |
| APR 1x | -0.28 | -1.84 | -2.14 | 1.41 |
| APR 2x | -0.85 | **-3.32** | **-3.71** | 0.57 |

APR, apramycin; 0.25x, 0.5x, 1x, 2x, apramycin concentration expressed as 0.25-, 0.5-, 1- or 2-fold the MIC

^a^A negative sign denotes a reduction of inoculum compared with time 0; values in bold are consistent with bactericidal activity

**Figure S2**. The difference between the mean starting inoculum and the mean viable cell count (Δlog_10_cfu/mL) of the 12 *A. baumannii* isolates over time after (a) *in vitro* exposure to 0.5xMIC mg/L of apramycin (APR) and 2 mg/L of colistin (COL) alone and in combination, (b) *in vitro* exposure to 1xMIC mg/L of apramycin (APR) and 2 mg/L of colistin (COL) alone and in combination and (c) *in vitro* exposure to 2xMIC mg/L of apramycin (APR) and 2 mg/L of colistin (COL) alone and in combination

(a) (b) (c)

**Figure S3**. The difference between the mean starting inoculum and the mean viable cell count (Δlog_10_cfu/mL) of the 12 *A. baumannii* isolates over time after (a) *in vitro* exposure to 0.5xMIC mg/L of apramycin (APR) and 30 mg/L of meropenem (MER) alone and in combination, (b) *in vitro* exposure to 1xMIC mg/L of apramycin (APR) and 30 mg/L of meropenem (MER) alone and in combination and (c) *in vitro* exposure to 2xMIC mg/L of apramycin (APR) and 30 mg/L of meropenem (MER) alone and in combination

(a) (b) (c)

**Figure S4**. The difference between the mean starting inoculum and the mean viable cell count (Δlog_10_cfu/mL) of the 12 *A. baumannii* isolates over time after (a) *in vitro* exposure to 0.5xMIC mg/L of apramycin (APR) and 3.5 mg/L of minocycline (MIN) alone and in combination, (b) *in vitro* exposure to 1xMIC mg/L of apramycin (APR) and 3.5 mg/L of minocycline (MIN) alone and in combination and (c) *in vitro* exposure to 2xMIC mg/L of apramycin (APR) and 3.5 mg/L of minocycline (MIN) alone and in combination

(a) (b) (c)

**Figure S5**. The difference between the mean starting inoculum and the mean viable cell count (Δlog_10_cfu/mL) of the 12 *A. baumannii* isolates over time after (a) *in vitro* exposure to 0.5xMIC mg/L of apramycin (APR) and 24 mg/L of sulbactam (SUL) alone and in combination, (b) *in vitro* exposure to 1xMIC mg/L of apramycin (APR) and 24 mg/L of sulbactam (SUL) alone and in combination and (c) *in vitro* exposure to 2xMIC mg/L of apramycin (APR) and 24 mg/L of sulbactam (SUL) alone and in combination

(a) (b) (c)
